# Trends in primary multidrug-resistant tuberculosis in the State of Rio de Janeiro: a retrospective study conducted during 2000-2019

**DOI:** 10.1590/0037-8682-0086-2021

**Published:** 2021-08-20

**Authors:** Marcela Bhering, Afrânio Kritski

**Affiliations:** 1 Universidade Federal do Rio de Janeiro, Faculdade de Medicina, Programa Acadêmico de Tuberculose, Rio de Janeiro, RJ, Brasil.; 2 Fundação Oswaldo Cruz, Escola Nacional de Saúde Pública Sergio Arouca, Rio de Janeiro, RJ, Brasil.

**Keywords:** Tuberculosis, Epidemiological monitoring, Drug resistance, Time-series studies

## Abstract

**INTRODUCTION:**

We analyzed the trends in primary multidrug-resistant tuberculosis (MDR-TB).

**METHODS:**

We performed a time series analysis of primary MDR-TB cases reported in the State of Rio de Janeiro (RJ) during 2000-2019. The annual percent change and the average annual percentage change (AAPC) were computed using joinpoint regression analysis.

**RESULTS:**

The percentage of cases increased from 7.69% in 2000 to 38.42% in 2018. We observed an upward trend during this period (AAPC = 9.4; 95% confidence interval 1.4-18.0, p < 0.001).

**CONCLUSIONS:**

The trend indicates the increasing occurrence of MDR-TB transmission sources in RJ during 2000-2019.

According to the World Health Organization estimates, in 2019, approximately 465,000 people developed rifampicin-resistant TB (RR-TB), of which 78% had multidrug-resistant TB (MDR-TB)^1^. MDR-TB is defined as resistance to rifampicin and isoniazid, which are two main drugs used to treat the disease^1^. Despite the treatments available, the overall success rate among these patients is 57%^1^. Among the estimated cases of MDR-TB in 2018, approximately 39% were reported, and 32% started treatment^2^.

In Brazil, after an average annual drop of 1% in incidence from 2009 to 2016^3^, there was an upward trend in TB cases during 2016-2019^1^. In 2017, among TB-MDR/RR cases, the proportion of cases wherein the patients were cured and had complete treatment was 55.7%^4^.

In 2018, the State of Rio de Janeiro (henceforth RJ), one of Brazil’s most developed states, had the highest mortality rate in the country, i.e., 4.3 per 100,000 inhabitants^4^, and the second-highest incidence rate among new TB cases, i.e., 63.3 per 100,000 inhabitants^3^. These data show that drug-resistant TB remains a threat to public health and an obstacle to meeting the Sustainable Development Goal (SDG) to end the TB epidemic by 2030 in RJ^5^.

Although the leading hypothesis for the increase in resistance cases is related to treatment failure (acquired drug resistance), the large gap in diagnosis and treatment increases the probability of the primary transmission of MDR-TB^6^. In the indexed literature, only few studies have analyzed the trends in primary MDR-TB. Thus, we conducted an original investigation at the sub-national level in Brazil.

The present study aimed to analyze the trends in MDR-TB cases with primary drug resistance in RJ. This ecological investigation was designed to identify the trends of a time series. The study included MDR-TB cases from RJ, notified through the Special Tuberculosis Treatment System (SITETB), an electronic information system of Brazil's Ministry of Health (MoH), which started treatment between January 2000 and December 2019. All patients had drug sensitivity testing (DST) resistant to rifampicin and isoniazid. SITETB is used for notifying and monitoring TB cases involving special treatments; this makes it impossible to use the standard TB regimen (2RHZE/4RH). Such cases may occur due to the occurrence of adverse reactions, toxicity, comorbidity, or drug resistance^7^. Primary resistance was defined as new MDR-TB patients who had never been treated for TB^8^. Cases of multidrug resistance were selected and classified as primary or acquired resistance according to the information recorded in the field “type of resistance” in SITETB.

We calculated the yearly proportion of MDR-TB cases with primary resistance (numerator: MDR-TB cases with primary resistance reported in the year; denominator: total MDR-TB cases reported in the year; multiplication factor: 100).

A joinpoint regression technique was used for the time-series analysis. This model checks whether a line with multiple segments is statistically superior to describe the temporal evolution of a dataset relative to a straight line or a line with fewer segments. Based on this logic, the model identifies the trends in the indicator (whether stationary, increasing, or decreasing) and the points where there is a change in the trend. This allows one to know the annual percent change (APC) and that for the entire period, called the average annual percent change (AAPC). For each detected trend, a 95% confidence interval (CI) and a 5% significance level were considered. The analysis was conducted using the Joinpoint Regression Program, version 4.8.0.1 (National Cancer Institute, Bethesda, MD, USA).

The study protocol was approved by the Hospital Universitário Clementino Fraga Filho Research Ethics Committee of the Federal University of Rio de Janeiro (CAAE 10126919.2.0000.5257).

During 2000-2019, a total of 2,790 cases of MDR-TB were reported in RJ, of which 533 (19.1%) were cases of primary MDR-TB. Among these, 66% were reported in the city of RJ, the capital of RJ. The proportion of MDR-TB cases with primary resistance among the reported MDR-TB cases ranged from 7.69% in 2000 to 38.42% in 2018 (Table 1).

**TABLE 1:** Number of MDR-TB cases, primary resistance, and acquired resistance and the proportion of primary MDR-TB cases from 2000 to 2019.

Year	Total cases of MDR-TB notified	Primary resistance	Acquired resistance	% Primary MDR-TB
2000	117	9	108	7.69
2001	122	12	110	9.84
2002	139	18	121	12.95
2003	113	10	103	8.85
2004	109	11	98	10.09
2005	104	12	92	11.54
2006	102	12	90	11.76
2007	98	19	79	19.39
2008	106	20	86	18.87
2009	102	22	80	21.57
2010	144	28	116	19.44
2011	150	28	122	18.67
2012	193	25	168	12.95
2013	173	16	157	9.25
2014	185	19	166	10.27
**2015**	154	37	117	**24.03**
**2016**	157	49	109	**31.21**
**2017**	181	57	124	**31.49**
**2018**	177	68	109	**38.42**
**2019**	164	61	103	**37.2**

MDR-TB: multidrug-resistant tuberculosis.

The regression model showed three temporal behaviors: between 2000 and 2009 (APC: 11.19; 95% CI 2.3-20.8, p < 0.001), increasing; between 2009 and 2013 (APC: −10.38; 95% CI 35.6-24.7, p = 0.5), stationary; and between 2013 and 2019 (APC: 21.85; 95% CI 11.9-32.7, p < 0.001), increasing. When analyzing the total period, a significant upward trend was observed in the proportion of MDR-TB cases with primary resistance (AAPC: 9.4; 95% CI 1.4-18.0, p <0.001) (Figure 1).

**FIGURE 1:**
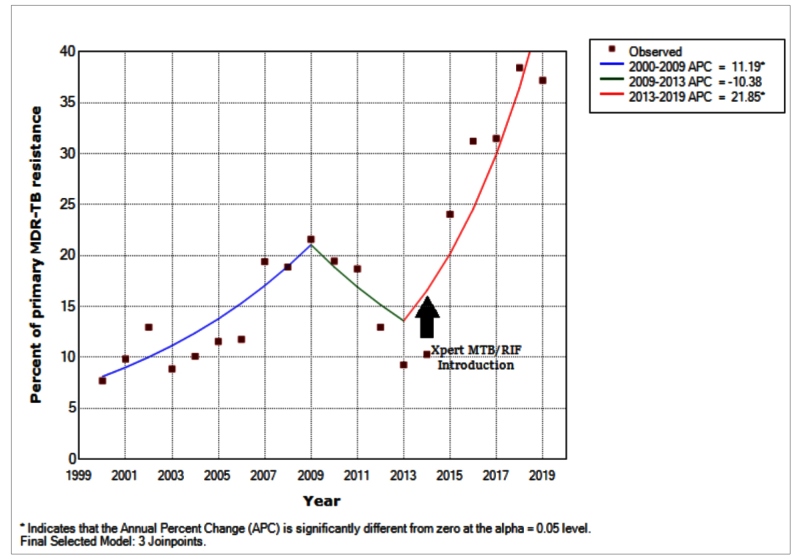
Trends in the cases of primary multidrug-resistant tuberculosis (MDR-TB) in the State of Rio de Janeiro during 2000-2019.

The aforementioned upward trend shows that there was failure to identify MDR-TB cases in RJ and, consequently, to control transmission and diagnosis. Early diagnosis, high-quality treatment for drug-sensitive and drug-resistant TB, effectiveness in implementing infection control, strengthening the rational use of medicines (standardized regimens and treatment adherence), and addressing individual risk factors and the social determinants of TB are the main strategies for preventing drug-resistant TB^8^.

In Brazil, until 2009, culture and DST performance depended on the investigated patients' clinical-epidemiological situation and the available laboratory resources. As of 2009, culture and DST have been recommended for all retreatment cases. As part of the End TB strategy, in 2014, the implementation of Xpert MTB/RIF began in Brazil, with the goal of lowering the time of diagnosis and identifying rifampin resistance at an early stage^9^. This contributed to the increase in the diagnosis of cases involving primary drug resistance. A study conducted in RJ and Manaus showed an increase in the bacteriological confirmation of pulmonary TB of 59% (95% CI = 31%-88%) as compared to that in sputum smear microscopy. The prevalence of rifampin resistance detected by Xpert was 3.3% among new cases and 7.4% among retreating patients^10^.

Despite the Ministry of Health's recommendation to offer a universal culture and DST, preliminary data from the MoH showed that, of the 9885 new pulmonary TB cases reported in 2019 in RJ, only 16.1% underwent culture and 35.5% underwent Xpert tests^4^.

There are many challenges to overcome in RJ. RJ, which is composed of 92 municipalities, is the third most populous state in Brazil. The city of Rio de Janeiro, which is the RJ capital, is the second most populous city in the country, with more than 6.7 million inhabitants; among them, 1.4 million inhabitants (22% of its population) live in informal settlements or slums^11^.

Slums are areas with poor infrastructure, lack of adequate sanitation, crowded populations, and poor personal hygiene and housing conditions, leaving the population more vulnerable to social inequalities and diseases^11^.

In the Rocinha slum, the TB incidence was 447.3 cases/100,000 inhabitants according to a study conducted among TB cases notified from 2004 to 2006^12^. Among the factors associated with a higher incidence of TB are a high frequency of more than two people per bedroom at home, population clusters, and high unemployment, in addition to low education levels and urbanization factors^12^.

Although TB is associated with socioeconomic factors, a recent study reported factors associated with treatment outcomes in patients with primary and acquired resistance. The study found significant differences in the demographic and clinical characteristics between the two groups. Proportionally, the group with primary drug resistance had predominantly female patients (46.4% vs. 33.5% in the acquired resistance group), Caucasians (47.3% vs. 34%), and those with schooling for ≥8 years (37.7% vs. 27.4%)^13^. These results emphasize that the primary cases of MDR-TB constitute another reservoir of transmission and are less related to lower socioeconomic levels^13^.

Another study in Peru reported a primary MDR-TB incidence rate of 6.3% (95% CI 4.4-8.3) in the general population, with no risk factors associated with MDR-TB (known exposure to a patient with TB whose treatment failed or the patient died or a patient affected with MDR-TB, immunosuppressive comorbidities, inmates, health workers, and alcohol or drug abuse). This result suggests that, in an endemic area, targeting MDR-TB patients based on the presence of risk factors is an insufficient intervention^14^.

Given that this is an ecological study, it was impossible to assess whether there are regions with a greater tendency for growth and whether they are associated with more significant population-dense areas and to determine the impact of demographic and clinical characteristics. This is an important limitation. Another limitation was that the study did not cover all TB cases notified in the same period. Hence, it was not possible to compare the trends in the proportion of MDR-TB cases relative to TB cases during the same period. However, the increase in the proportion of primary MDR-TB cases during the study period reported in RJ suggests that the transmission of drug-resistant strains among the general population is of great concern. 

 We conclude that the upward trend during the 2000-2019 period indicates an increase in primary MDR-TB cases in transmission sources, which, in turn, makes it even more difficult to eliminate MDR-TB in RJ and highlights the importance of Xpert MTB/RIF to improve the diagnosis of primary drug resistance, as well as the urgency to expand strategies to reduce MDR-TB transmission.
